# Characterization of Cytomegalovirus Lung Infection in Non-HIV Infected Children

**DOI:** 10.3390/v6052038

**Published:** 2014-05-07

**Authors:** Sonia M. Restrepo-Gualteros, Lina E. Jaramillo-Barberi, Monica Gonzalez-Santos, Carlos E. Rodriguez-Martinez, Geovanny F. Perez, Maria J. Gutierrez, Gustavo Nino

**Affiliations:** 1 Division of Pediatric Pulmonology, Fundacion Hospital La Misericordia, Bogota 111411, Colombia; E-Mail: sm.restrepo@uniandes.edu.co; 2 Department of Pediatrics, School of Medicine, Universidad de los Andes, Fundacion Santa Fe de Bogota, Bogota 110111, Colombia; 3 Department of Pathology, School of Medicine, Universidad Nacional de Colombia, Bogota 111321, Colombia; E-Mail: millito59@hotmail.com; 4 Department of Pathology, Fundación Hospital La Misericordia, Bogota 111411, Colombia; 5 Department of Pediatrics, School of Medicine, Universidad Nacional de Colombia, Bogota 111321, Colombia; E-Mails: mikg376@gmail.com (M.G.-S.); carlos2671@gmail.com (C.E.R.-M.); 6 Department of Pediatric Pulmonology and Pediatric Critical Care Medicine, School of Medicine, Universidad El Bosque, Bogota 110111, Colombia; 7 Research Unit, Military Hospital of Colombia, Bogota 111321, Colombia; 8 Division of Pulmonary and Sleep Medicine, Children’s National Medical Center, Washington, DC 20010, USA; E-Mail: gperez@childrensnational.org; 9 Division of Pediatric Rheumatology, Allergy and Immunology, Pennsylvania State University College of Medicine, Hershey, PA 17033, USA; E-Mail: mgutierrez@hmc.psu.edu; 10 Department of Integrative Systems Biology, Center for Genetic Medicine Research, Children’s National Medical Center, George Washington University, Washington, DC 20010, USA

**Keywords:** CMV, lung, pneumonia, children, ground glass

## Abstract

Cytomegalovirus (CMV) is a prevalent pathogen in the immunocompromised host and invasive pneumonia is a feared complication of the virus in this population. In this pediatric case series we characterized CMV lung infection in 15 non-HIV infected children (median age 3 years; IQR 0.2–4.9 years), using current molecular and imaging diagnostic modalities, in combination with respiratory signs and symptoms. The most prominent clinical and laboratory findings included cough (100%), hypoxemia (100%), diffuse adventitious breath sounds (100%) and increased respiratory effort (93%). All patients had abnormal lung images characterized by ground glass opacity/consolidation in 80% of cases. CMV was detected in the lung either by CMV PCR in bronchoalveolar lavage (82% detection rate) or histology/immunohistochemistry in lung biopsy (100% detection rate). CMV caused respiratory failure in 47% of children infected and the overall mortality rate was 13.3%. Conclusion: CMV pneumonia is a potential lethal disease in non-HIV infected children that requires a high-index of suspicion. Common clinical and radiological patterns such as hypoxemia, diffuse adventitious lung sounds and ground-glass pulmonary opacities may allow early identification of CMV lung infection in the pediatric population, which may lead to prompt initiation of antiviral therapy and better clinical outcomes.

## 1. Introduction

Cytomegalovirus (CMV) is one of the most important causes of opportunistic lung infection in the pediatric population [1]. Risk factors include human immunodeficiency virus (HIV) infection and immunocompromised status after bone marrow/stem cell transplantation (BMT/SCT) and solid organ transplantation [2]. Despite being a life threatening condition, if diagnosis is made in a timely manner, CMV pneumonia can potentially be cured with appropriate antiviral therapy (*i.e.*, ganciclovir) [3]. Unfortunately, the clinical diagnosis of CMV lung infection is challenging in children and often requires a high-index of suspicion, especially in non-HIV infected cases and patients not severely immunocompromised [4].

Most of the literature about CMV lung infection in children focuses on the epidemiology of this condition in the pediatric immunocompromised population [1]. There are few reports that address the specific clinical and pulmonary imaging findings that may suggest this difficult diagnosis. Smith *et al.* in 1977 described the clinical respiratory findings and radiological appearance of CMV pulmonary infection in children using plain X-ray films [5]. This seminal work enhanced the awareness of the atypical course of CMV infection in the lungs, often described as “CMV pneumonitis” [5], which includes non-specific pulmonary findings such as interstitial patterns with increased bronchopulmonary markings and bronchiolar disease (diffuse air trapping) [5]. Interestingly, there has been a dramatic change in both the epidemiology of CMV infection and diagnostic techniques available for its diagnosis in children over the last 30 years [6]. Now-a-days we have an increasing population of non-HIV infected infants and children who have received different modalities of immunomodulators (*i.e.*, steroids) for various conditions including BMT/SCT, solid-organ transplant and systemic autoimmune/inflammatory disorders that put them at risk for CMV lung infection [7]. In addition, better molecular diagnostics (*i.e.*, CMV qualitative real-time PCR) as well as great progress in performing pediatric bronchoscopy and detailed lung imaging (*i.e.*, high-resolution computerized tomography, HRCT) have improved our ability to detect CMV pulmonary infection, re-defining the concept of CMV pneumonia, which is now known to occur in children that are not severely immunocompromised [8].

The goal of this article is to characterize CMV lung infection in non-HIV infected children using current molecular and imaging diagnostic modalities, in combination with traditional respiratory signs and symptoms. To this end, we present a case series of 15 children with CMV lung infection focusing on their clinical presentation, radiological patterns, bronchoscopic findings and the molecular approaches used. Our results highlight that the diagnosis of CMV pneumonia in children is challenging but the common clinical and radiological patterns such as hypoxemia, diffuse adventitious lung sounds and ground-glass pulmonary opacities, can provide critical clues to allow early identification of CMV lung infection in the pediatric population.

## 2. Experimental

### 2.1. Patients

Fifteen cases of CMV pulmonary infection in non-HIV pediatric patients were studied. Neonates, infants and children (0–14 years) of both genders were included. All patients were seen at La Misericordia Children’s Hospital in Bogota, Colombia, which is the largest University-based Pediatric Hospital in the country. Cases were collected between January 2010 and November 2013. Children with HIV infection were not included. No other medical conditions were considered as exclusion criteria. CMV lung infection cases were defined as positive pulmonary detection of CMV by polymerase chain reaction (PCR) or immunofluorescence (lung biopsy) and clinical evidence of lung involvement on exam and from radiographic images of the chest.

### 2.2. Clinical Assessment

Clinical variables were obtained by electronic medical record review (EMR) and included symptoms involving the respiratory system, such as cough and signs/symptoms indicative of increased breathing effort including tachypnea, retractions, nasal flaring and/or dyspnea. Other respiratory variables included abnormal breath sounds (rales, wheezing, rhonchus, *etc.*), pulsoximetry values and the need/duration for mechanical ventilation. General symptoms like fever and weight loss were also recorded. Duration of disease, time to diagnosis and length of hospitalization were included as well.

### 2.3. Diagnostic Studies

Clinical specimens used for the diagnosis of CMV lung infection included serum and bronchoalveolar lavage (BAL). CMV detection was performed using real time PCR methodology (Light Cycler CMV Quantitative Kit, Roche Diagnostics, Indianapolis, IN, USA), which is routinely conducted in our institution. CMV viral culture is not available and not routinely performed in our hospital. BAL was obtained via fiberoptic bronchoscopy from the right middle lobe, lingula or affected site according to ERS guidelines [9]. Neutrophilia in BAL was defined as >3% based on prior studies and bronchoscopy guidelines for children [9]. In five patients, open lung biopsy was also performed and the lung tissue was submitted for histopathological analysis to identify typical CMV features (*i.e.*, inclusion bodies) and for immunohistochemistry studies using CMV-specific antibodies. Chest radiographs and high resolution CT scans (HRCT) were performed in all patients using pediatric protocols and general anesthesia if appropriate. Radiological findings were analyzed using standardized Fleischner Society [10] terminology by pediatric radiologists and independently by at least two pediatric pulmonologists. Other tests performed included CMV serology (serum IgM by enzyme-linked immunosorbent assay) and follow-up studies for CMV infection including serum CMV PCR/viral load measurements as well as ongoing monitoring of hemogram and liver function testing for drug toxicity.

## 3. Results

The baseline characteristics of the study subjects are presented in Table 1. The median age of the 15 children with CMV lung infection was 3 years (IQR 0.2−4.9 years.), which most likely reflects the population treated by the authors and not necessarily the group that is more likely to be affected by CMV. The majority were immunocompromised (13/15, 87%) according to immune cell counts, immunoglobulin levels or concomitant use of immunosuppressant agents (steroids). Specifically, children on systemic steroids (11/15, 73%) had low values for age of either IgG (6/11, 54%), absolute lymphocyte counts (8/11, 73%), absolute neutrophil counts (1/11, 9%), or total CD4 counts (2/11, 18%). Other important risk factors included malnutrition, hypogammaglobulinemia, hematologic malignancy and bone marrow/solid organ transplantation (Table 1). All transplant patients included had prior record of negative serology for CMV (donor and recipient).

**Table 1:** Baseline characteristics for pediatric subjects.

N	15
Male, n (%)	8 (53)
Age (year), median (IQR)	3 (0.25–4.9)
**Comorbidity **	
Immunocompromised, n (%)	13/15 (87)
Systemic steroid use, n (%)	11 (73)
Hypogammaglobulinemia	8 (53)
Malnutrition, n (%)	6 (40)
Acute leukemia, n (%)	3 (20)
Autoimmune disease, n (%)	2 (13)
Allogeneic bone marrow/stem cell transplant, n (%)	2 (13)
Renal transplant, n (%)	1 (6)

*IQR*, interquartile range.

### 3.1. Clinical Characterization of CMV Lung Infection

Clinical presentation of CMV lung infection was variable among pediatric subjects (Table 2). The median time of symptom onset was 14 days (IQR 3–20 days) with a median time to diagnosis of 26 days (IQR 11–37 days). All subjects presented with cough and 93% had increased respiratory effort/dyspnea as reported by the parent and/or patient. Other non-specific symptoms included fever and weight loss, which were present in 10 (67%) and 8 (53%) subjects respectively. The two most prominent abnormal laboratory findings were anemia (53%) and abnormal liver function (47%), which has been previously reported as a hallmark of acute CMV infection [11]. Other common hematologic abnormalities included thrombocytopenia (40%), leukocytosis (40%) and leukopenia (27%).

The clinical respiratory assessment was abnormal in all children with CMV pulmonary infection (Table 3). Physical examination findings included diffuse abnormal breath sounds (100% of patients) including wheezing (80%), rales (73%) and rhonchus (67%). All subjects showed hypoxemia at presentation and 47% required mechanical ventilation for respiratory failure. The median duration of mechanical ventilation was eight days (IQR 7–26 days). Overall mortality rate was 13% (*n* = 2). One subject died due to severe CMV infection and one due to massive central nervous system bleeding, secondary to severe thrombocytopenia associated with systemic lupus erythematosus.

**Table 2:** Clinical presentation of Cytomegalovirus (CMV) lung infection in children.

Duration	
Onset of symptoms (days), median (IQR)	14 (3–20)
Time to diagnosis (days), median (IQR)	26 (11–37)
**Presenting symptoms**	
Cough, n (%)	15 (100)
Increased breathing effort, n (%)	14 (93)
Fever, n (%)	10 (67)
Weight loss, n (%)	8 (53)
**Laboratory findings**	
Anemia, n (%)	8 (53)
Thrombocytopenia, n (%)	6 (40)
Leukocytosis, n (%)	6 (40)
Leukocytopenia, n (%)	4 (27)
Abnormal liver function, n (%)	7 (47)

*IQR*, interquartile range.

**Table 3:** Respiratory Manifestations of CMV lung infection in children.

Clinical variables	
Abnormal lung sounds, n (%)	15 (100)
Wheezing, n (%)	12 (80)
Rales, n (%)	11 (73)
Rhonchus, n (%)	10 (67)
Hypoxemia, n (%)	15 (100)
Mechanical ventilation (MV), n (%)	7 (47)
Days of MV, median (IQR)	8 (7–26)
Death, n (%)	2 (13)
**Radiological variables**	
Abnormal lung images, n (%)	15 (100)
Ground-glass opacity, n (%)	12 (80)
Consolidation, n (%)	12 (80)
Atelectasis, n (%)	6 (40)
Air trapping, n (%)	5 (33)
Nodular pattern, n (%)	3 (20)
Reticular pattern, n (%)	1 (6)
Tree-in-bud pattern, n (%)	1 (6)

*IQR*, interquartile range.

### 3.2. Radiological Findings in CMV Lung Infection

**Figure 1:**
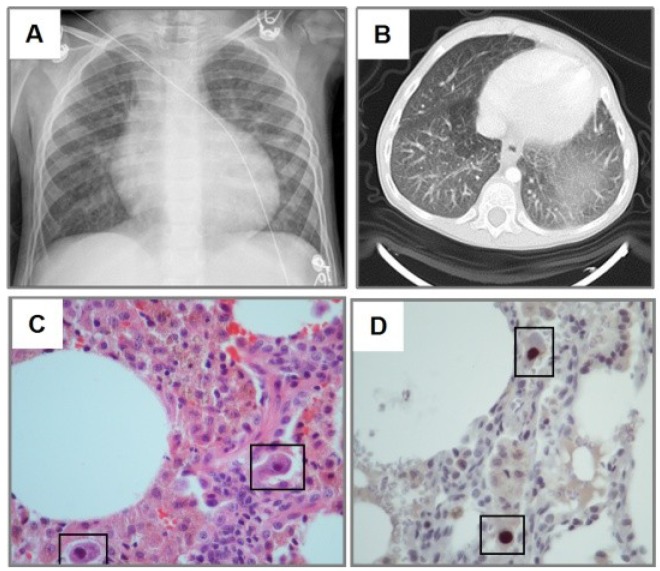
Cytomegalovirus (CMV) lung infection after steroid therapy. Three year old girl treated with systemic corticosteroid for autoimmune lymphoproliferative syndrome (ALPS). Chest-X-ray (CXR) shows diffuse haziness more prominent in the left base (**A**), which correspond to ground-glass images in CT scan (**B**). H&E staining revealed monocyte infiltration with cytomegalic changes in lung biopsy (black squares in panel **C**). Immunohistochemical detection of CMV (brown staining in **D**) showed typical CMV inclusion bodies (squares). Slides shown with 100× magnification.

**Figure 2:**
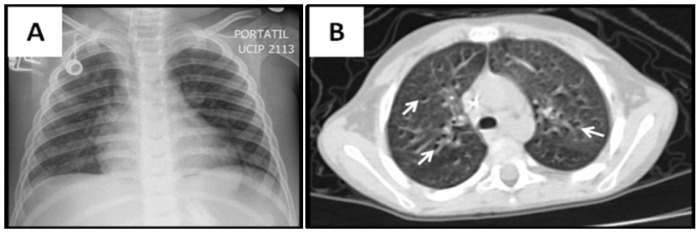
Ground-glass pattern in CMV lung infection after stem cell transplant (SCT). Four year old boy with acute lymphoid leukemia presenting with hypoxemia and bilateral rales after allogeneic SCT. Diffuse haziness is seen in CXR (**A**), and CT scan (**B**). There are interspersed airway lumen marks known as “dark bronchus” signs indicating ground-glass pattern (arrows in **B**).

**Figure 3:**
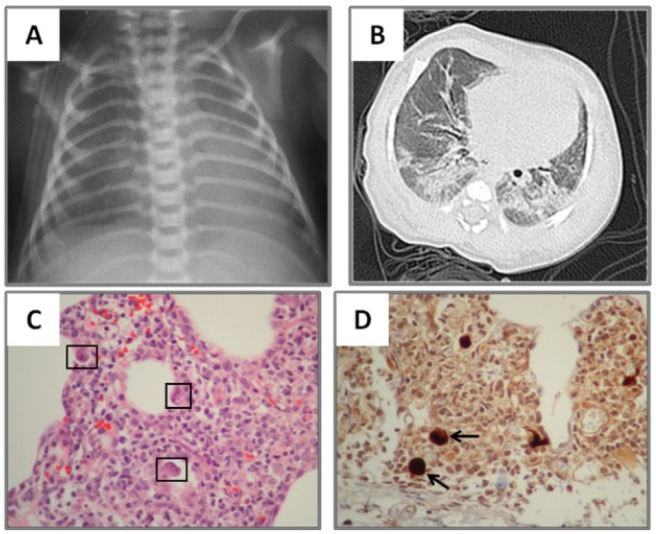
Ground-glass/Consolidation pattern in neonatal CMV lung infection. One month old term baby with lung haziness in CXR (**A**). CT scan revealed diffuse ground-glass and consolidations in both lung bases (**B**). Cytomegalovirus polymerase chain reaction) (CMV PCR) was (+) in serum but (−) in bronchoalveolar lavage. Lung biopsy revealed dense monocyte infiltration with cytomegalic changes (black squares in panel **C**). Immunohistochemical detection of CMV in the lung (brown staining in **D**) revealed characteristic inclusion bodies (arrows). Slides shown with 40× magnification.

**Figure 4:**
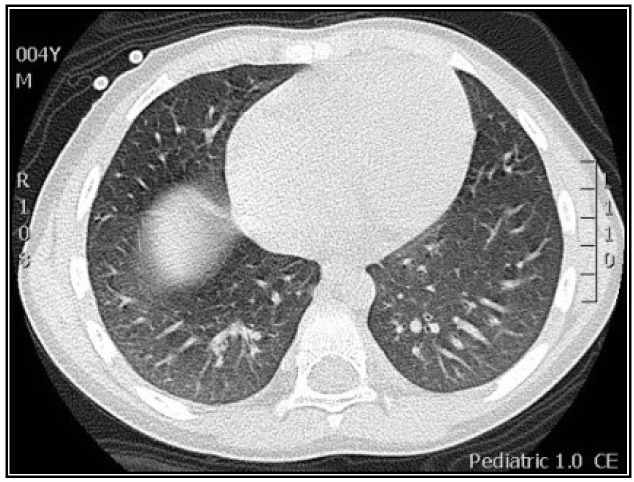
Ground-glass pattern in CMV lung infection detected with high resolution CT scan (HRCT). Four year old boy with hypoxemia and diffuse rales/wheezing after bone marrow transplant. Plain CXR revealed non-specific mild increased lung markings. HRCT identified subtle ground-glass pattern in both bases. CMV diagnosis was confirmed by (+) PCR in bronchoalveolar lavage.

**Figure 5:**
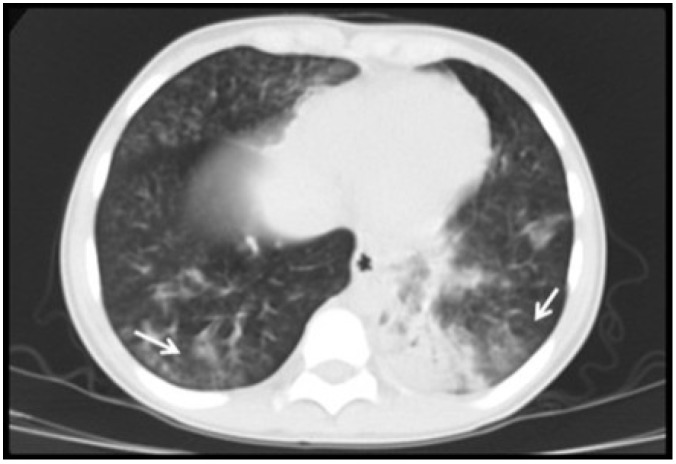
Consolidation pattern in CMV lung infection. Fourteen year old boy with history of renal transplant. A retrocardial consolidation is seen in CT scan along with scattered opacities with lower density resembling ground glass pattern (arrows). Bronchoalveolar lavage of the left lower lobe revealed a (+) PCR for CMV.

All patients had abnormal lung images. The most prominent radiological findings on plain chest radiographs and chest CT were consolidation and ground-glass opacity (80%) (Figure 1, Figure 2, Figure 3, Figure 4 and Figure 5), indicating partial filling of alveolar air spaces, interstitial thickening and/or partial collapse of the alveoli during CMV pulmonary infection [10]. The result of these alveolar lesions is lung haziness in chest-X-ray (CXR) and ground-glass opacity on the CT scan [10,12]. In our pediatric case series, ground-glass opacity and consolidations were diffuse (Figure 1, Figure 2, Figure 3, Figure 4 and Figure 5) and co-existed in eight patients (53%). In cases where CXR showed minimal abnormalities, the use of high-resolution CT scan (HRCT) enhanced the ability to see ground-glass opacity and consolidations (Figure 4). Atelectasis was present in 40% of cases and air trapping in 33%. Other radiological lung patterns were rare and included nodular (20%), reticular (6%) and tree-in-bud pattern (6%) (Table 3).

### 3.3. Diagnostic and Treatment Approaches of CMV Lung Infection in Children

**Figure 6:**
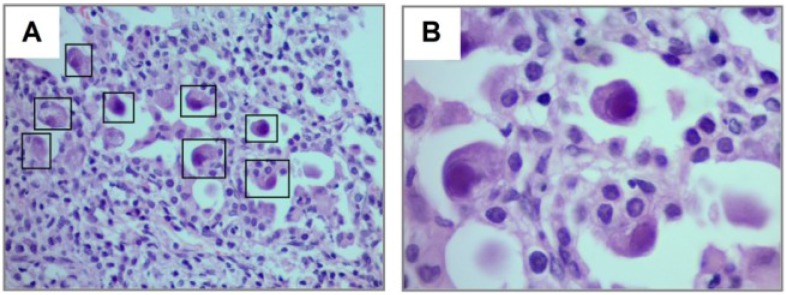
Cytopathic changes in CMV pulmonary infection after steroid therapy**.** Thirteen year old boy treated with systemic corticosteroid for bronchiolitis obliterans and severe asthma presenting with acute hypoxemia and diffuse rales/wheezing. Lung biopsy revealed diffuse cytopathic changes in pneumocytes infected by CMV (squares in **A** and magnified image in **B**) including cellular enlargement and abnormal nucleus. Figures show H&E staining with magnification 40× (**A**) and 100× (**B**).

The diagnostic approaches included a combination of CMV IgM serology, CMV serum PCR/viral load, bronchoscopy with bronchoalveolar lavage (BAL) with quantitative CMV PCR and lung biopsy (Table 4). The most common diagnostic modalities used were CMV serum PCR/viral load (14/15), which yielded a positive result in 50% of cases with a median CMV viral load of 268 copies/μL (IQR 20–20,000 copies/μL), and BAL (12/15) which gave positive CMV PCR in 82% of cases (Table 4). Most children with CMV lung infection had neutrophilia in BAL cellularity (92%) (Table 4). Lung biopsy was performed in five subjects and CMV was confirmed by histopathology and immunohistochemical detection (Figure 1, Figure 3 and Figure 6). A number of patients (14/15) were treated with IV ganciclovir during 14 to 21 days at dose of (5 mg/kg every12 h), which is the standard CMV treatment in our institution. Patients were followed for an average period of 2–3 weeks after therapy. One patient was not treated because of full resolution of CMV infection before starting antiviral therapy. Four subjects also received oral valganciclovir treatment after IV ganciclovir. Antiviral therapy was effective in 13/14 patients (one patient died due to severe CMV infection) and no significant side-effects were reported.

**Table 4:** Diagnostic and treatment approaches of CMV lung infection in children.

Intervention	
**Bronchoalveolar lavage (BAL), n (%)**	**12 (80)**
CMV PCR (+) in BAL, n (%)	9/11(82)
Neutrophilia in BAL, n (%)	11/12(92)
**Lung biopsy, n (%)**	**5 (33)**
Biopsy confirmed CMV, n (%)	5/5 (100)
**CMV serum PCR viral load, n (%)**	**14/15 (93)**
CMV PCR viral load (+) in serum	7/14 (50)
CMV copies/μL, median (IQR)	268 (20–20,000)
**CMV serology, n (%)**	**5/15 (33)**
CMV IgM (+) in serum	4/5 (80)
**CMV treatment, n (%)**	**14/15 (93)**
Ganciclovir, n (%)	14/14 (100)
Valganciclovir, n (%)	4/14

***I****QR*, interquartile range; *PCR*, polymerase chain reaction.

## 4. Discussion

CMV is a herpes virus that can produce life-threatening pulmonary infections in immunocompromised hosts [4]. Although effective antiviral therapy for CMV is available [11,13], timely diagnosis remains a major challenge for this condition, particularly in the pediatric population where CMV often presents with atypical patterns of lung infection [5,14]. The diagnosis of CMV pneumonia can be even more difficult in situations when there is no high-index of suspicion, for instance, in patients that do not have an underlying immunodeficiency syndrome (*i.e.*, HIV-infected patients). Indeed, a detailed clinical characterization of CMV lung infection in this age group is critical to select patients that may warrant invasive procedures to obtain lung samples (*i.e.*, pediatric bronchoscopy or open lung biopsy) and may benefit from prompt CMV therapy before confirmatory testing is available. Given the paucity of data about the clinical features of CMV pulmonary infection in children, this paper aims to fill this gap in the literature detailing the clinical and radiological features of this condition in a case series (*n* = 15) of non-HIV infected pediatric patients with pulmonary CMV infection. Our data illustrates that despite significant variability in the clinical presentation, there are specific features consistently present in pediatric CMV pulmonary infection, including hypoxemia, diffuse adventitious sounds and ground-glass consolidation pattern in lung CT scan, which together may offer crucial clues in the diagnosis of this condition in children.

As previously reported in the adult literature, we found that the majority of children that developed CMV pneumonia were immunocompromised (87%) and 73% of patients were on systemic steroid therapy for different conditions including asthma, systemic lupus erythematous, leukemia treatment, renal transplant and allogeneic bone marrow/stem cell transplant. In addition, 8/15 of the subjects (53%) had hypogammaglobulinemia, which was mostly secondary to steroid treatment and/or malnutrition in our case series. These risk factors have been previously reported in the literature [15], and reflect the need to have proper humoral and cell-mediated immunity to clear CMV infection in the lungs [3,16,17]. During the study period we did not identify children with HIV and pulmonary CMV in our institution. This may reflect the population treated by the authors and not the overall trend of CMV infections in pediatric HIV. In this regard, it is important to emphasize that children with HIV may have different patterns of CMV lung disease. For instance, HIV itself is often associated with non-malignant lymphocytic infiltrative disorders, including nonspecific interstitial pneumonitis and lymphocytic interstitial pneumonitis (LIP) [18], which could potentially change the radiological appearance and the clinical manifestations of opportunistic lung infections like CMV. In addition, HIV patients might have more extra-pulmonary manifestations of CMV (retinitis and hepatitis) as well as other opportunistic lung pathogens such as Pneumocystis Jirovecci [19] that may lead to more severe disease and worse prognosis.

In terms of the clinical presentation and respiratory compromise, the onset of symptoms had a mean time of 14 days however there was considerable variation ranging from three days in a patient that required early mechanical ventilatory support, and up to three weeks in one child with nonspecific symptoms, a feature that has previously labeled CMV as an unpredictable respiratory infection [5]. Overall there was also great heterogeneity in the severity of the respiratory compromise. While one patient had self-limited clinical course that did not required therapy with ganciclovir, another had severe CMV infection and died despite antiviral therapy. In contrast, there was a consistent homogenous pattern of initial respiratory symptoms/signs that included a combination of cough, hypoxemia, increased breathing effort (*i.e.*, retractions or dyspnea) and diffuse abnormalities in lung auscultation, which were present in virtually all cases (Table 3). The diffuse adventitious lung sounds identified were described as either wheezing, rales and/or rhonchus, which suggests variable degrees of involvement of lung parenchyma and conductive airways during CMV infection, compatible with what is generally described in CMV lung pathology [20]. In association with respiratory symptoms, about half of the patients had constitutional manifestations (fever or weight loss) and accompanying laboratory abnormalities that included anemia (53%), thrombocytopenia (40%), leukocytosis (40%), leukopenia (27%) and elevated liver enzymes (47%). These abnormal laboratory findings are in overall agreement with prior reports of CMV lung infection in children [11,21].

One of the most important findings of our study was the current difficulty/delay in establishing the diagnosis of pediatric CMV lung infection. There was a median time to diagnosis of 26 days (IQR 11–37 days), which could be explained by the low clinical suspicion in the initial management of these non-HIV infected neonates and children. Indeed, relatively prompt diagnosis was obtained in those individuals with BMT, SMC and bone marrow transplantation (Figure 2, Figure 4 and Figure 5), which are well-known risk factors for CMV and therefore bronchoscopy/BAL was performed early. On the other hand individuals without underlying immunodeficiency (*i.e.*, steroid-dependent asthma; Figure 6) or malnutrition were subject to longer time to diagnosis. Another factor that contributed to CMV diagnosis delay was the turnover time for the molecular studies. The CMV molecular diagnostic approach was performed using several modalities, including CMV viral load in peripheral blood, CMV qualitative PCR amplification in bronchoalveolar lavage (BAL) fluid and IgG serology for CMV. Prior reports have been found in which measurements of CMV viral DNA done in samples of BAL or sputum are better to make a diagnosis of CMV pneumonia, compared with the gold standard demonstration of cytomegalic inclusions in lung tissue [22]. Honda, J., *et al.* studied 363 CMV adult patients with 882 samples of sputum, BAL, peripheral blood and urine, showing a positive predictive value (PPV) and negative predictive value (NPV) of 100% and 98.8% for BAL samples, 95.5% and 99.7% for sputum samples; and a sensitivity 90.9% and specificity 100% for BAL and 95.5% and 99.7% for sputum [22]. In our pediatric case series, CMV PCR in BAL was obtained in 11/15 and was positive in 82% of the cases (9/11); which may reflect the technical difficulties of obtaining a proper BAL specimen in the pediatric population, particularly in neonates [9]. Interestingly, most children with CMV lung infection had neutrophilia instead of lymphocytosis in BAL cellularity, which has been described in the adult literature [7,23]. The later may be attributed to bacterial superinfection, immunosuppression (affecting lymphocyte function and proliferation) or differences in the airway immune response to CMV in the pediatric population. In addition to bronchoscopy, four patients also underwent lung biopsy, two of these had PCR negative for CMV in BAL and the other two had a PCR positive in BAL but biopsy was performed due to additional concerns about other pulmonary infectious/inflammatory processes (*i.e.*, persistent hypoxemia and alveolar hemorrhage). There was one patient who did not undergo a flexible bronchoscopy with BAL before open lung biopsy. In all five cases, lung biopsy confirmed the diagnosis of CMV, demonstrating cytopathic changes (Figure 6) and positive immunohistochemistry (Figure 1 and Figure 3). Additional pathological findings in lung biopsies were diffuse monocytic infiltration (Figure 1, Figure 3 and Figure 6) alveolar hemorrhage and pulmonary vascular disease in a patient with history of hemosiderosis and pulmonary hypertension. Because not all patients had the same CMV testing done (*i.e.*, viral load) we could not do correlations of laboratory values with clinical parameters or the severity of CMV lung infection. 

The initial imaging diagnostic approach consisted of chest radiography and later chest CT to further characterize the parenchymal involvement and to help in the decision making for further testing such as flexible bronchoscopy and/or lung biopsy. One hundred percent of patients had abnormalities on radiological exams; the most frequent findings were consolidation, usually compromising the dependent lung regions (bi-basal consolidation; Figure 3) and ground-glass opacities caused by the partial displacement of air due to filling of alveolar spaces, interstitial thickening and/or partial collapse of alveoli leading to enhanced small airway lumen marks, which is a radiological sign known as “dark bronchus” [10] (Figure 2). The ground glass/consolidation pattern has been previously identified in adults with CMV infection [24,25]. These radiological findings underlie the clinical presentation of CMV lung infection in our pediatric series that included increased respiratory effort, abnormal breath sounds and hypoxemia secondary to diminished alveolo-capillar gas diffusion and abnormal ventilator (V)/ perfusion (Q) match in the lungs [25,26]. Importantly, CT scan was superior to CXR in detailing the radiological pattern of CMV lung infection in our pediatric case series. This is in agreement with Smith *et al.* who previously reported children with CMV pulmonary disease having minimal non-specific abnormalities in CXR [5]. In this context, it is noteworthy to mention that newer CT scan lung modalities (high-resolution) are now being proposed to diagnose different pediatric pulmonary conditions and thus avoid open lung biopsies [27]. For instance, some types of children’s interstitial lung disease (chILD) such as neuroendocrine cell hyperplasia in infancy (NEHI), have specific patterns of disease in CT scan that may be sufficient to make a diagnosis when combined with a specific set of clinical features [27]. Based on our case series, we propose a group of clinical criteria (Table 5) based on risk factors (immunosuppression), symptoms/signs and lung imaging that when present must raise concern for CMV pulmonary infection in children and should prompt further confirmatory investigation (*i.e.*, bronchoscopy) or empiric therapy depending on the clinical situation.

**Table 5:** Key features of CMV lung infection in non-HIV infected children.

**Immunosupression**
Systemic steroid use
Malnutrition
Hypogammaglobulinemia
Hematologic malignancy
Post-transplantation
**Clinical**
Cough
Increased breathing effort
Hypoxemia
Diffuse adventitious lung sounds (*i.e.*, rales, wheezing)
**Imaging**
Ground-glass opacity/consolidation
**Bronchoscopy**
CMV PCR in BAL

*BAL*, Bronchoalveolar lavage.

## 5. Conclusions

In summary, CMV infection of the lung is a prevalent condition in the immunocompromised host with a variable clinical presentation. Clinical clues that should prompt suspicion of CMV pneumonia in this population are the presence of respiratory symptoms and abnormal lung exam. The initial diagnostic approach should include a chest-X ray which can shows signs consistent with alveolar air displacement, however these changes can be subtle and easily missed on interpretation, thus making a chest CT a better radiological tool to evaluation the lung involvement in the immunocompromised host. The best test to confirm the diagnosis is a CMV PCR performed in BAL, bronchoscopy. The prompt identification of CMV lung infection in children without underlying immunodeficiency syndrome (*i.e.*, non-HIV infected) requires a high-index of suspicion based on common respiratory sign/symptoms/images (Table 5) and is crucial to institute early antiviral therapy, which can significantly impact the clinical outcome of neonates and children with this potentially life-threatening infection.
